# Prevalence and Risk Factors of Hepatitis C Virus Infection in Amol City, North of Iran: A Population-Based Study (2008-2011)

**DOI:** 10.5812/hepatmon.13313

**Published:** 2013-12-07

**Authors:** Farhad Zamani, Masoudreza Sohrabi, Hossein Poustchi, Hossein Keyvani, Fatemeh Sima Saeedian, Hossein Ajdarkosh, Mahood Khoonsari, Gholamreza Hemmasi, Maziar Moradilakeh, Nima Motamed, Masoumeh Maadi

**Affiliations:** 1 Gastrointestinal and Liver Diseases Research Center (GILDRC), Iran University of Medical Sciences, Tehran, IR Iran; 2 Liver and Pancreatobiliary Diseases Research Center, Digestive Diseases Research Institute, Tehran University of Medical Sciences, Tehran,IR Iran; 3 Department of Virology, Tehran University of Medical Sciences, Tehran, IR Iran

**Keywords:** Hepacivirus, Epidemiology, Polymerase Chain Reaction, Urban Population, Rural Population

## Abstract

**Background:**

Hepatitis C Virus (HCV) infection is one of the most important causes of chronic liver disease and related problems in the world .There are few population-based studies on the prevalence and risk factors of hepatitis C infection in Iran, which could not provide enough information. Moreover, the prevalence and risk factors of hepatitis C infection are not similar in all parts of Iran.

**Objectives:**

The aim of this survey was to determine the prevalence and risk factors of HCV infection in the general population of the city of Amol, north of Iran.

**Patients and Methods:**

This was a population-based study. Using a cluster sampling approach, 6145 individuals of both genders and different ages were involved from general population of urban and rural areas of Amol, The inclusion criteria were Iranian nationality, willing to participate in the study, and lifelong residence in Amol city and surrounding areas. Anti-hepatitis C antibody was measured by a third generation of ELISA. The positive results were confirmed by Recombinant Immuno Blot Assay (RIBA) and quantitative HCV-RNA polymerase chain reaction (PCR) tests. Potential risk factors of HCV transmission were recorded.

**Results:**

The mean age of participants was 42.70 ± 17.10 years. Of these participants, 57.2% (n = 3483) were male. Anti-HCV antibody was positive in 12 individuals from which five were RIBA positive. Three of these subjects were PCR positive. The prevalence of HCV was more predominant among males than females. The common risk factors among the study subjects included history of minor or major surgery (34.7%), unsterile punctures (21.2%), history of traditional phlebotomy (5.8%), and history of hepatitis among close relatives (5.7%). In univariate regression analysis, unsterile punctures and history of infection in family members were associated with HCV infection.

**Conclusions:**

We confirm that in Amol city and surrounding areas, the prevalence of true HCV infection is 0.05%, which is lower than that previously reported from Iran.

## 1. Background

Hepatitis C Virus (HCV) infection is a leading cause of chronic liver disease and related problems in the world (1-3). The world-wide prevalence of hepatitis C infection is about 3% according to a World Health Organization (WHO) report with over 130 million infected cases (1, 2). Despite obligatory blood screening for HCV since 1990 along with development of advanced diagnostic methods, the prevalence of HCV infection is roughly stable or even increased in developing countries (4-8). Therefore, determination of distribution and risk factors of HCV infection is an important issue towards understanding the main ways of HCV transmission, which in turn, may assist health organizations to plan better preventative policies. 

There are few population-based studies in Iran conducted on the prevalence and risk factors of HCV infection. The majority of studies, however, were focused on specific high-risk groups which might not represent actual rate of infection in general population (9). The first report on HCV prevalence in Iran was published in 1994 and revealed that 0.3% of healthy blood donors had seroprevalence of anti- HCV-Ab (10). However, based on recent reports, the HCV-Ab positive rate in Iran is 0.13% - 0.89% (11-13). The prevalence of HCV infection in specific populations in Iran are as high as 26.4% among hemodialysis patients, 11.9% among nursing home residents, about 52% among intravenous drug user, and 15-75% among patients with blood disorders such as hemophilia and thalassemia (13-16) . According to previous reports, male gender, history of transfusion, being intravenous drug user, and interfamilial HCV infection are common risk factors related to HCV infection (11, 13, 16). 

HCV infection rate appears to be rising in this territory and further epidemiological studies may assist in the prevention, control, and subsequent treatment of HCV infections. Due to lack of enough information regarding prevalence of HCV infection in different parts of Iran, more population–based studies are necessary. 

## 2. Objectives

Therefore, the present study endeavors to provide more up-to-date information derived from a population-based survey designed for the city of Amol, in northern Iran.

## 3. Patients and Methods

### 3.1. Study Area and Population

Amol is one of the largest cities in north- center of Iran, located in Mazandaran province, with a population of approximately 300,000 inhabitants. The population of this territory is homogeneous equally distributed in rural and urban areas, the fact that strengthens reliability of this study. From 2008, the Gastro Intestinal and Liver Disease Research Center (GILDRC) has conducted a multidisciplinary study on general population of Amol and surrounding areas. In this study the prevalence and risk factors of viral hepatitis C have been assessed.

The general population of urban and rural areas of Amol, northern Iran, was studied. Urban areas refer to urbanized or municipal areas; rural areas refer to non-urban or agricultural areas. The inclusion criteria were Iranian nationality, lifelong residence in the Amol vicinity, and willingness to participate in the study. Subjects with age over 10 years were included in this study.

### 3.2. Sample of Study 

The study performed as part of a health cohort in Amol from 2008. Clustered random sampling was used. Health Centers are referred to as ‘clusters’ in this study. Family registration at health centers was used, which facilitated random selection of subjects. By a multiple-stage sampling method, we selected 107 Health Centers with average 30 household sizes. The samples were collected from each Health Center based on each cluster population. Total 6420 subjects were involved in this study. 

### 3.3. Data Collection and Laboratory Tests

In collaboration with Health Centers, the research team scheduled a face-to-face interview with all eligible participants in the clinics from May 2008 to March 2011. If the study subject was not present in the first scheduled meeting, a second appointment was scheduled. If the subjects missed three consecutive appointments at the clinics, the research team would offer to set up an appointment in their homes. Individuals who refused or were unable to participate were excluded and replaced by other subjects chosen from the same cluster. All participants were asked to complete a questionnaire providing information regarding age (number of years according to their identification documents), gender (male or female), marital status (single or married, based on own subjects' responses), occupation (high risk jobs such as medical staffs and nursery home workers), literacy level (five level: illiterate ,< 6 years, ≤ 12 years, ≤ graduate, ≥ masters), blood transfusion history, history of surgery (either minor or major operation), viral hepatitis (hepatitis C, hepatitis B), narcotic drug abuse (habitual narcotic or drug abuse more than once per week), smoking (active smoker or not), tattooing and piercing (presence of figures , signs, or scars), cupping and blood-letting (yes or no; based on subjects' response to question).

Then, all eligible study participants were referred to Haraz Research Center, a branch of GILDRC, to complete clinical and laboratory evaluations. A 30-milliliter (ml) blood sample was taken from each subject. Five milliliter was used for detection of viral markers using ELISA (Enzyme-Linked Immuno-Sorbent Assay) and remained amount was stored in a -80-degree centigrade freezer. Acon kits (Acon Lab., San Diego, CA, 92121, USA) were used to test hepatitis C markers. HCV–Ab positive samples were checked using RIBA (Recombinant Immuno Blot Assay) followed by qualitative HCV-RNA polymerase chain reaction. A false positive is defined as both RIBA and PCR negative. A resolved infection is defined as both ELISA and RIBA positive but PCR negative. True infection is definitive when all three tests are positive.

### 3.4. Statistics Analysis

The results were analyzed by the Statistical Package for the Social Sciences (SPSS) for Windows (Version 16.0, Chicago, IL, USA). The prevalence of data was assessed by descriptive analysis. Variables were analyzed by Chi-Square (in some situations, Fisher Exact test were applied), Student's T-Test as appropriate. Univariate forward LR logistic regression was used for evaluation of potential risk factors associated with HCV infection. All variables (gender, marriage status; history of IDU, imprisoned, transfusion, surgery, transfusions, tattooing, traditional phlebotomy, unsterile puncture, and family history of HCV infection in one relative) were included in this model. A P-value less than 0.05 were considered as significant. 

### 3.5. Ethics

The participants were informed about the project and written consent was obtained from all participants before data collection. All data were securely stored in the study database. The study protocol and consent form were approved by the Board of Ethics of the GILDRC.

## 4. Results

In this study, 6145 participants were recruited, so the response rate was 96% . Of these, 57.2% (n = 3483) of cases were male. The mean age of participants was 42.70 ± 17.10 years, and roughly half (48.9%) of the subjects were from rural areas. The mean ages of urban and rural subjects were 43.1 ± 16.10 and 42.25 ± 17.40 years, respectively. Also, 51.3% and 63.1% of urban and rural participants were male, respectively. Table 1 shows all qualitative variables and frequencies. 

Of 6145 participants, ELISA anti-HCV antibody was positive in 12 (0.2%) individuals, of which 66.6% (n = 8) were male. Half of these patients were from urban areas (M/F = 3/3). However, the RIBA test was positive in five (0.08%) male participants; finally only three persons (0.05%) were PCR positive. In this context, three subjects showed true infection, two subjects resolved infection, and seven subjects false positive infection. 

**Table 1.** Demographic Data of Study Subjects in Relation to Anti-HCV- Ab ELISA Positive result

Variables	No. (%)
**Subject interviewed**	6145 (-)
**Male**	3515 (57.2)
**Rural**	3005 (48.9)
**Currently married**	4719 (76.8)
**History of surgery**	2133 (34.7)
**History of unsterile puncture**	1303 (21.2)
**History of blood transfusion**	362 (5.9)
**History of traditional phlebotomy**	357 (5.8)
**Family history of hepatitis**	351 (5.7)
**History of Tattooing**	197 (3.2)
**History of imprisonment**	86 (1.4)
**HBs Ag**	74 (1.2)
**High risk Occupational**	61 (1)
**History of addiction**	30 (0.5)
**History of unsafe sexual contact**	30 (0.5)
**History of IDU**	25 (0.4)

In this study, the risk factors of hepatitis C infection among all participants were also evaluated. The different risk factors are summarized in Table 2. The main risk factor was history of surgery (34.7%), followed by punctures, traditional phlebotomy (cupping and bloodletting), and a family history of hepatitis. In present study, history of punctures (P = 0.024) and history of family infection (P = 0.001) were associated with prevalence of HCV infection. Univariate odds ratios for main risk factors were computed by logistic regression. Unsterile punctures (OR = 6.142; CI 95% 1.296 – 29.112) showed a correlation with HCV infection. Although an association was found between a family history of HCV infection in one relative (OR = 2.466; CI 95% 0.410 – 14.59), it was not considered significant. However, no association was found between HCV- Ab and surgery, transfusions, tattooing, traditional phlebotomy, and gender. 

**Table 2.** The Results of RIBA Test in ELISA Positive Patients

Variables	Anti-HCV-Ab Elisa Positive, No. (%)	Anti-HCV-Ab RIBA Positive, No. (%)
**Gender**		
Male	8 (66.6)	5 (100)
Female	4 (33.4)	0
**Marriage status**		
Married	10 (83.4)	1 (20)
Single	2 (16.6)	4 (80)
**Age groups, y**		
≤ 30	4 (33.3)	4 (80)
30-39	1 (8.3)	1 (20)
40-49	4 (33.3)	0
50-59	3 (25)	0
≥ 60	0	0
**Location**		
Urban	6 (50)	1 (20)
Rural	6 (50)	4 (80)
**Education **		
Illiterate	2 (16.6)	0
< 6 y	2 (16.6)	2 (40)
≤ 12 y	6 (50)	2 (40)
≤ Graduate diploma	2 (16.6)	1 (20)
≥ Master (MS)	0	0
**Transfusion**	2 (16.6)	2 (40)
**Addiction and Intravenous Drug User**	1 (8.3)	1 (20)
**Tattooing**	1 (8.3)	1 (20)
**Punctures**	4 (33.3)	2 (40)
**Imprisonment**	0	0
**Surgery**	4 (33.3)	2 (40)
**Unsafe sexual contact**	1 (8.3)	0
**High risk jobs**	0	0
**History of hepatitis in a member of Family **	5 (41.6)	0
**Traditional Phlebotomy**	3 (25)	2 (40)

## 5. Discussion

Hepatitis C infection raised dramatically in recent decades in the Middle Eastern region. It might be related to changes in the main route of transmission in this territory. Epidemiological studies, particularly in areas with the lack of adequate information, could help to prevent chronic HCV infection problems. Therefore, in present study we tried to identify HCV infection rate in the city of Amol, north of Iran, and compare it with previous reports. 

In this survey, seroprevalence (RIBA positive) of HCV infection was 0.08% and only three cases (0.05%) were PCR positive (true infection). This rate is much lower than previous findings and reports from different countries, including Iran. The seroprevalence of HCV infection (RIBA test) in Iran is estimated at about 0.20% in general population (14-16). Infection rate in different provinces of Iran is not similar .The prevalence of HCV infection in studies conducted in different geographical areas of Iran was 0.3% to 0.86% (10, 11, 13, 14). The city of Amol is located in north-center of Iran in Mazandaran province, immediately adjacent to Golestan province in north-east of Iran where infection rate is significantly higher compared to the result of present study (0.08% vs. 1.0%). As mentioned previously, the majority of studies on HCV prevalence were conducted on special groups. For instance, Amini – Kafabad et al. reported a prevalence of 0.13% of HCV infection in a study among blood donors during a 3-year period (17). The reason for the ambiguity in HCV infection rates in different parts of Iran is not clear but could be due to life style, population density, and public education about hepatitis C infection. Also, frequent travelling to the countries with high rate infection might be accounted. 

The evaluation of HCV infection risk factors among participants was another important objective of the present study. Due to limited number of infected patients, evaluation of risk factors might not be significant in present study. Typically the transmission of HCV occurs through infected blood and blood products as well as contaminated devices, such as needle. Intravenous drug use (IDU) is an important route of HCV transmission, and previous reports have shown a history of IDU in many of the patients (11, 13). However, the study did not confirm this finding and the authors have no definitive explanation for these results, but possibly might be due to a low rate of IDU in this study compared to previous studies. Also family support might influence on this finding. Furthermore, harm-reduction policies including educating patients and their families, providing sterile needles, and isolation/separation of IDU patients by local healthcare system may influence on the results. Analysis revealed that non-sterile punctures, as part of blood contamination, and a history of family member infection augment risk of contamination. There is limited data on risk of HCV transmission among family members. Hepatitis C infection may be spread by routes similar to hepatitis B. In this context, sharing contaminated objects such as needles as well as non-adherence to strict health code recommendations play major roles in the transmission of HCV. Furthermore, unsafe sexual contact, tattooing, and imprisonment were reported in previous studies (13, 14). However, the results of present study were not in accordance with results obtained from those results. This might be related to low prevalence of HCV infection in this region along with limited number of subjects who were previously incarcerated.

In this study, the prevalence of HCV-Ab in both rural and urban areas was equal while the RIBA and ELISA positive were more common in rural areas (4 vs. 1). To the authors’ knowledge, there are limited studies comparing the rate of infection between rural and urban areas. In the present study, HCV infection rate in rural areas is significantly higher than that in urban areas corresponding with previous reports (13). This difference might be explained by socioeconomic, lifestyle, and educational inequalities in two areas. 

Male gender was reported as an independent predictive factor for HCV infection in different studies (13, 16, 17). Interestingly, in the present study all of the HCV infected cases were male. This could indicate the higher prevalence of high-risk behaviors among men compared to women. 

 It should be noted that approximately 75% of HCV- Ab positive subjects were prone to chronic hepatitis C; also it was reported that 70% of RIBA HCV-Ab positive were also HCV- RNA- positive (8, 15, 16). In this study, 5 subjects were RIBA positive among which three cases (60%) had chronic hepatitis C. This result might be due to the limited number of HCV positive subjects in this study.

The study had some limitation. First, due to low prevalence and dispersion of risk factors we could not present an appropriate judgment. Second, based on the subjects' memory, recall bias was a prone for limitation; this study faced also with responder bias due to some private and sensitized questions. Third, we had used clustered random sampling for selection of participants but we did not consider cluster analysis, although because of low rate of positive cases this could not influence on results. 

In conclusion, the present study revealed that prevalence of HCV in general population of Amol and surrounding areas was much lower than declared in reports already published on Iran.
